# Cα-H···O=C hydrogen bonds contribute to the specificity of RGD cell-adhesion interactions

**DOI:** 10.1186/1472-6807-5-4

**Published:** 2005-02-14

**Authors:** Jordi Bella, Martin J Humphries

**Affiliations:** 1 Wellcome Trust Centre for Cell Matrix Research, Faculty of Life Sciences, University of Manchester

## Abstract

**Background:**

The Arg-Gly-Asp (RGD) cell adhesion sequence occurs in several extracellular matrix molecules known to interact with integrin cell-surface receptors. Recently published crystal structures of the extracellular regions of two integrins in complex with peptides containing or mimicking the RGD sequence have identified the Arg and Asp residues as key specificity determinants for integrin recognition, through hydrogen bonding and metal coordination interactions. The central Gly residue also appears to be in close contact with the integrin surface in these structures.

**Results:**

When hydrogen atoms are modelled on the central Gly residue with standard stereochemistry, the interaction between this residue and a carbonyl group in the integrin surface shows all the hallmarks of Cα-H···O=C hydrogen bonding, as seen in the collagen triple helix and in many crystal structures of small organic molecules. Moreover, molecular dynamic simulations of the docking of RGD-containing fragments on integrin surfaces support the occurrence of these interactions. There appears to be an array of four weak and conventional hydrogen bonds lining up the RGD residues with main chain carbonyl groups in the integrin surface.

**Conclusions:**

The occurrence of weak Cα-H···O=C hydrogen bonds in the RGD-integrin interaction highlights the importance of the conserved Gly residue in the RGD motif and its contribution to integrin-ligand binding specificity. Our analysis shows how weak hydrogen bonds may also play important biological roles by contributing to the specificity of macromolecular recognition.

## Background

The Arg-Gly-Asp (RGD) sequence is one of the most easily recognised motifs in molecular biology [1]. Discovered in fibronectin in 1984 [2], this tripeptide appears to be conserved in the cell attachment sites of many proteins from the extracellular matrix (ECM). The later discovery that RGD is recognised by members of the integrin family of cell surface receptors [3], confirmed the central role of RGD and suggested that its presence in a protein sequence might be indicative of cell-adhesion functionality [4]. Integrins are ubiquitously expressed heterodimer cell surface molecules that act as receptors for ECM molecules and other cell-surface adhesins. Through these cell-matrix and cell-cell interactions integrins control diverse cell functions such as adhesion, shape, growth, differentiation and mobility, and therefore contribute to important physiological processes such as development, immune responses and cancer [5]. Integrins are complex signalling engines: their extracellular domains interact with the ECM while their cytoplasmic tails interact with the cytoskeleton and other intracellular signalling molecules. Current hypotheses suggest that conformational changes resulting from these interactions enable integrins to transmit signals across the membrane in both directions. Recent advances in the structural biology of several integrin domains and their interactions with ligands have begun to define possible working scenarios for the signalling mechanisms [6-13].

**Figure 1:**
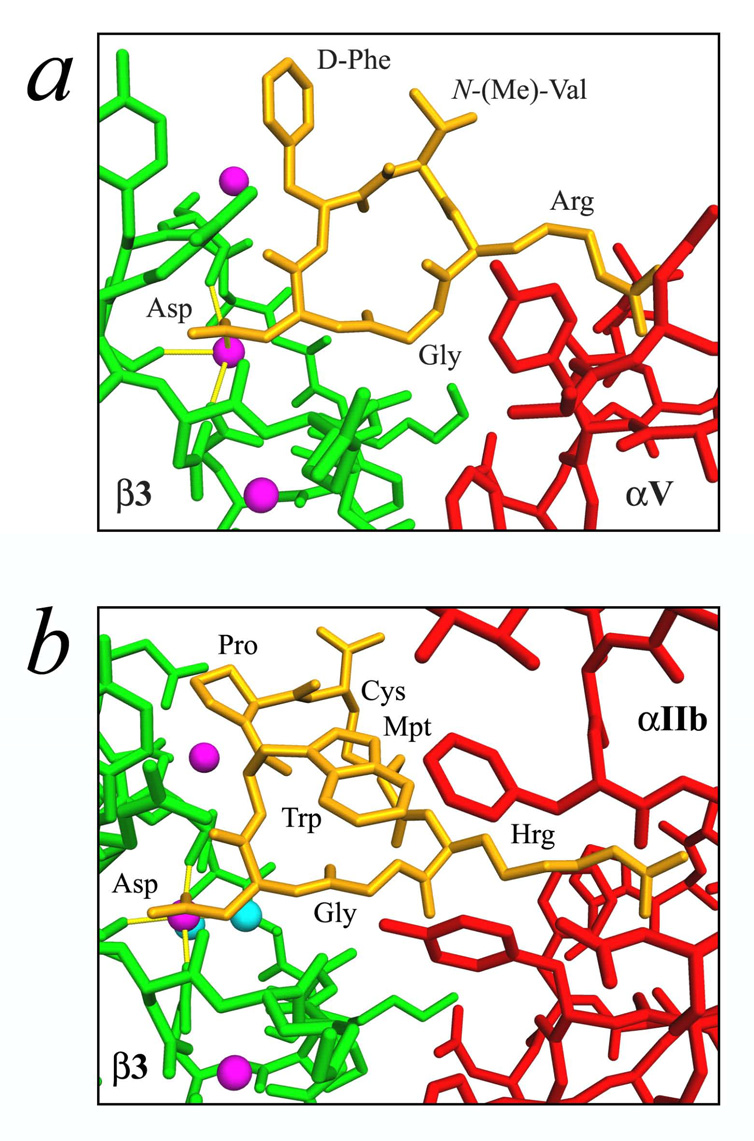
Binding of peptide ligands to the integrin surfaces. **(*a*) **Detail of the crystal structure of the extracellular region of αVβ3 integrin in complex with the cyclic pentapeptide Arg-Gly-Asp-D-Phe-N(Me)-Val [8]. The peptide (orange), sits across the interface between the αV (red) and β3 (green) integrin subunits, but only the three amino acids from the RGD triad make significant contact with the integrin surface. The Asp residue completes the coordination of one of the three Mn^2+ ^ions (purple spheres) at the top of the β3 subunit. **(*b*) **Detail of the crystal structure of the extracellular region of αIIbβ3 integrin in complex with the cyclic peptide eptifibatide [12], showing very similar interactions. Hrg and Mpt indicate L-homoarginine and β-mercaptopropionic acid residues, respectively. Due to higher resolution, water molecules (cyan spheres) are seen in this structure to complete the coordination of the metal ions. Other colours as in panel *a*. Both figures have been prepared using *SETOR *[45].

As a consequence of their role in so many fundamental processes, integrin defects have been implicated in many common diseases, from cancer to pathogen invasion. An ability to block a particular integrin-ligand interaction may be a possible route to the control of certain pathological states, hence it is not surprising that some integrins have become attractive targets for drug design. Understanding the molecular bases of the interaction of integrins with their ligands is therefore essential for effective protein-based design of inhibitors or activators of their function. A milestone was reached in 2002 with the determination of the crystal structure of the extracellular segment of αVβ3 integrin in complex with a cyclic peptide containing the prototypical RGD sequence [8]. In that structure, the amino acids defining the RGD sequence are seen to establish specific interactions with corresponding residues in the integrin heterodimer surface, spanning the interface between the αV and β3 subunits (Figure 1a). Very recently, another landmark paper has reported several crystal structures of the extracellular region of the fibrinogen-binding integrin αIIbβ3 [12]. In addition to providing an improved picture of the allosteric basis of integrin signal transmission, this new set of structures shows the molecular details of the interaction between the αIIbβ3 RGD-binding site and various ligand mimetics (Figure 1b). These interactions are remarkably consistent with those previously observed in the complex between the αVβ3 integrin fragment and the cyclic RGD peptide (*c*RGD) [8].

At first glance, two interactions consistently seen in these crystal structures appear to be key in defining the specific molecular recognition between the RGD sequence in an integrin ligand and the surface of its integrin receptor: the Asp residue of the RGD triad completes the coordination of a divalent metal ion bound to the β subunit, while the Arg side chain extends in the opposite direction to form salt-bridge hydrogen bonds with one or two Asp residues in the α subunit. These two specific interactions or their equivalent are seen both in the *c*RGD-αVβ3 structure and in the structures of αIIbβ3 in complex with ligand-mimetics (Figure 1). There are no significant hydrophobic "pockets" or exosites contributing to the binding specificity. For example, a large fraction of the *c*RGD peptide does not make any contact with the αVβ3 integrin surface (Figure 1a). In the broader context of RGD-containing ligands and their integrin receptors, it would seem that these interactions are mainly electrostatic and that the two charged residues in the RGD sequence are necessary and sufficient for attachment [14].

**Figure 2:**
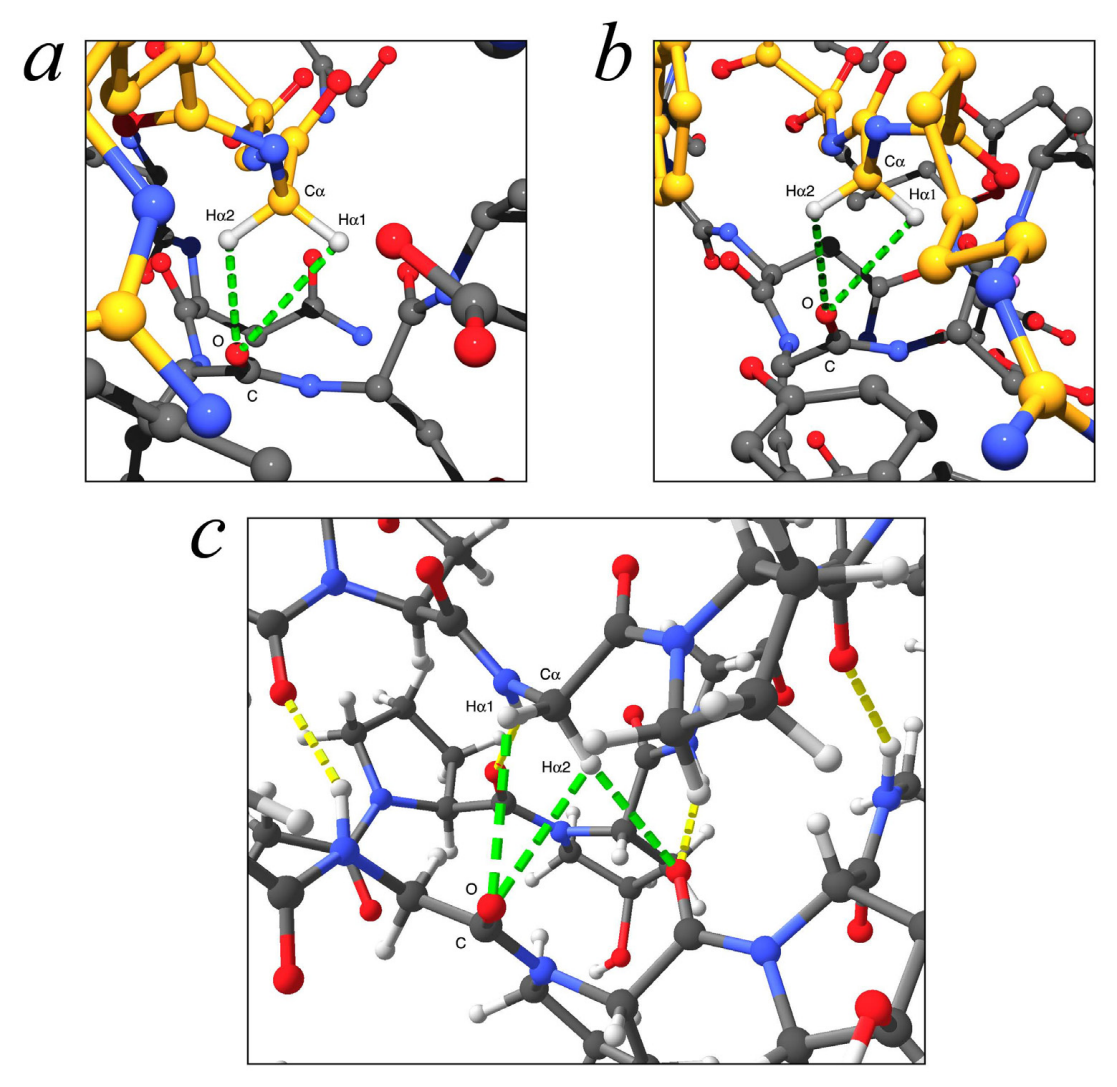
Geometry of Gly-Cα···O=C interactions after Gly α-hydrogen atoms are placed in their stereochemically predicted positions for: **(*a*) **the *c*RGD-αVβ3 crystal structure, and **(*b*) **the EFB-αIIbβ3 crystal structure. Atoms are colour coded as follows: oxygen, red; nitrogen, blue; hydrogen, white; metal, purple; carbon from integrin in grey; and carbon from the *c*RGD and EFB peptides in orange. Cα-H···O hydrogen bonds are shown as green dashed lines. **(*c*) **Hydrogen bonding in the collagen triple helix [15]. Conventional hydrogen bonds are shown in yellow, Cα-H···O hydrogen bonds in green. The two Hα atoms in collagen Gly residues participate in a bifurcated and three-centred hydrogen bonding configuration. Naming of Gly-Hα atoms follows the convention that Hα1 is equivalent to Hα in L-amino acids.

What about the central Gly residue? In their analysis of the *c*RGD-αVβ3 crystal structure, Xiong *et al. *report that the Gly central residue makes several hydrophobic interactions with the integrin surface, including a contact with the carbonyl oxygen of residue Arg216 in the β3 integrin subunit [8]. Such contact between the Gly methylene group and a main-chain carbonyl oxygen is also observed in the crystal structure of αIIbβ3 in complex with the peptidomimetic eptifibatide (EFB) [12] (Figure 1b), which is a cyclic heptapeptide containing a *homo*Arg-Gly-Asp sequence. The particular geometry of these contacts is strikingly reminiscent of a motif previously described in the collagen triple helix: a hydrogen bonding arrangement where the α-carbon of the Gly residue acts as hydrogen bonding donor in Cα-H···O=C interactions (Figure 2) [15].

So-called "weak" hydrogen bonds, such as those between carbon and oxygen atoms, have been traditionally neglected in descriptions of three-dimensional structures of macromolecules. Yet, C-H···O hydrogen bonds are ubiquitous in protein structures: virtually every conventional N-H···O=C hydrogen bond in every β-sheet in every determined protein structure carries a companion Cα-H···O=C interaction [16,17]. This applies to both parallel and antiparallel β-sheets, and exactly the same topology is also observed in the collagen triple helix [15]. For collagen and the β-sheet structures, the occurrence of Cα-H···O=C interactions is indicative of a very tight fit between the molecules involved, a close-packed structure in which all groups participate in some form of hydrogen bonding interaction.

How important are Cα-H···O=C and other weak hydrogen bonds in shaping the three-dimensional structure of proteins and macromolecular complexes? The subject has stimulated considerable debate (see [18] and [19] for reviews), although theoretical studies leave no doubt about the cohesive nature of these interactions [20-23]. With a strength approximately one-half of that from conventional hydrogen bonds, it seems reasonable to assume that the large numbers of weak hydrogen bonds detected in proteins may contribute to their stability. Furthermore, several biochemical functions have been linked to specific C-H···O hydrogen bonds, where position is more important that numbers. One example is the Gly-X-X-X-Gly motif, known to favour helix-helix interactions in membrane [24] and soluble proteins [25]*via *position-specific Cα-H···O=C hydrogen bonds. Another is the proposed role of C-H···O hydrogen bonds from cytosine and thymine bases to amino acid side chains during DNA-protein recognition [26]. Weak C-H···O hydrogen bonds have also been surveyed at protein-protein interfaces [27], and have been reported to play specific roles in catalysis [28], and in substrate and inhibitor recognition [29-32]. Recently, a server to identify weak hydrogen bonding interactions in protein structures has been made publicly available [33].

The functional occurrence of weak C-H···O hydrogen bonds in protein-ligand, protein-protein, and protein-DNA recognition suggests that their presence should be examined in detail in the structures of macromolecules with biomedical or biotechnological interest. Their potential should not be neglected in rational drug design approaches [31]. With this in mind, we present here an analysis of possible Gly-Cα-H···O=C interactions between RGD motifs and the RGD-binding sites from the αVβ3 and αIIbβ3 crystal structures. We conclude that the mutual geometry of the interaction is consistent with Cα-H···O=C hydrogen bonding. We discuss the implications of these hydrogen bonds for the cell adhesion interactions between integrins and their RGD-containing ligands.

## Results and discussion

Building standard-geometry Hα atoms on the Gly central residues of the *c*RGD and EFB peptides produces the geometric arrangements shown in Figure 2, clearly reminiscent of the hydrogen bonding pattern previously described in the collagen triple helix (Figure 2c). The metrics of these Gly-carbonyl contacts are shown in Table 1. The Cα···O distances in the *c*RGD-αVβ3 and EFB-αIIbβ3 structures appear to be longer than the mean Cα···O distance in collagen, but are well within the observed range in crystal structures of small organic molecules (see below). Both Hα atoms from the collagen Gly residues are in hydrogen bonding position (Cα–Hα···O > 90°), and their Cα-Hα···O=C hydrogen bonds adopt a three-centred and bifurcated configuration (Figure 2c and [15]), that is not seen in the integrin structures. Nevertheless, the central Gly residues in the *c*RGD and EFB peptides appear to have one and two Hα atoms respectively in hydrogen bonding position to the carbonyl group of Arg216, a residue on the surface of the β3 subunit and directly at the interface with the αV and αIIb subunits.

An obvious *caveat *to this analysis comes from the moderate resolution of the *c*RGD-αVβ3 and EFB-αIIbβ3 crystal structures (3.2 Å and 2.9 Å respectively). Positional errors inevitable at that resolution may affect the precision of the fitting of the *c*RGD and EFB peptides and the accuracy of the hydrogen bonding geometries for both weak and strong hydrogen bonds. For example, a close look at the salt-bridge interactions between the Arg guanidinium group from the *c*RGD peptide and two Asp side chains on the αV integrin surface (Asp150 and Asp218), shows less than "ideal" hydrogen bonding orientation, especially for Asp150 (not shown). Yet, the accumulated knowledge of hydrogen bonding geometries in high-resolution crystal structures and their significant variability leaves no doubt about the existence of these strong hydrogen bonds and their contribution to the specificity of binding.

**Figure 3:**
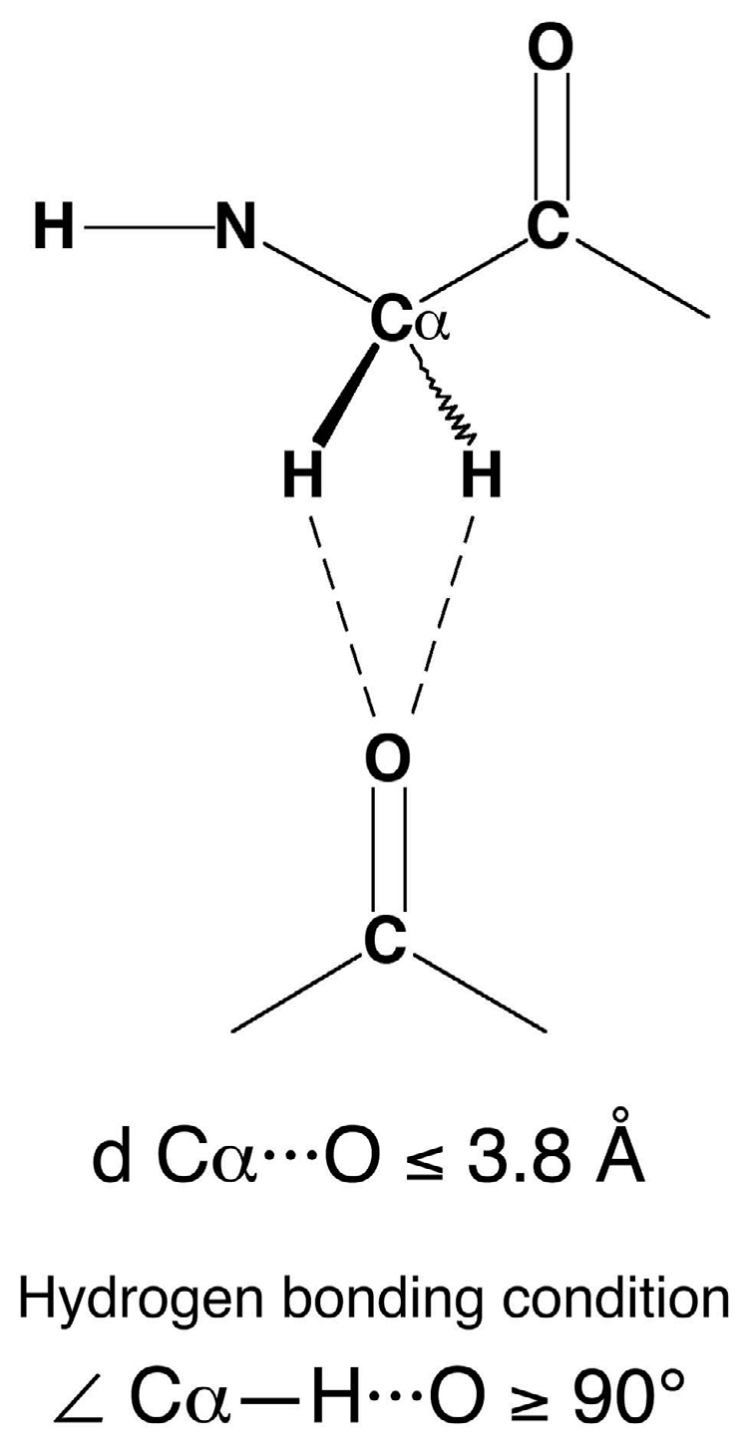
Fragments used in searches for non-bonded interactions in the Cambridge Structural Database [44]. Å 3.8 Å Cα···O distance cutoff was applied in all searches. A hydrogen atom was deemed to be in hydrogen bonding position if the angle Cα-H···O ≥ 90°. Separate searches were conducted for the bifurcated hydrogen bond (both angles ≥ 90°), single Cα-H···O=C hydrogen bond (one angle ≥ 90°, the other < 90°) and no hydrogen bonds (both angles < 90°).

**Figure 4:**
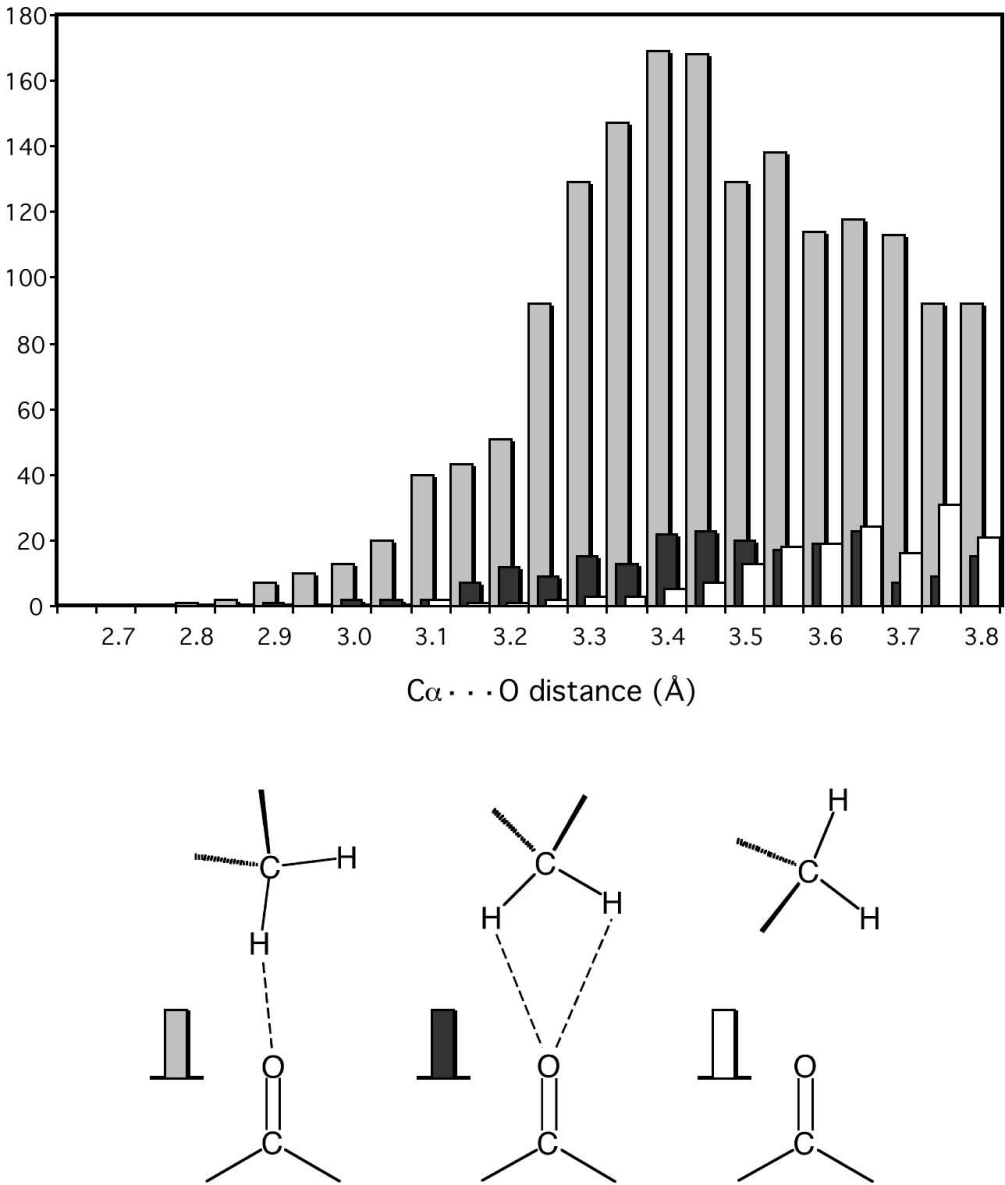
Distribution of Cα···O distances in the Cambridge Structural Database structures (July 2003 release), containing the motif depicted in Figure 2. Three cases are considered: single Cα-H···O hydrogen bond (light grey), bifurcated hydrogen bond (dark grey), and no hydrogen bond (white). The single hydrogen bonded case clearly dominates with 1688 hits overall, for 218 of the bifurcated case and 166 hits for the no hydrogen bond case. The maximum in the single hydrogen bond distribution around 3.4 Å suggests that value as the Cα···O hydrogen bonding distance, although a significantly large number of interactions can be still classified as hydrogen bonds at the longer Cα···O distances.

**Figure 5:**
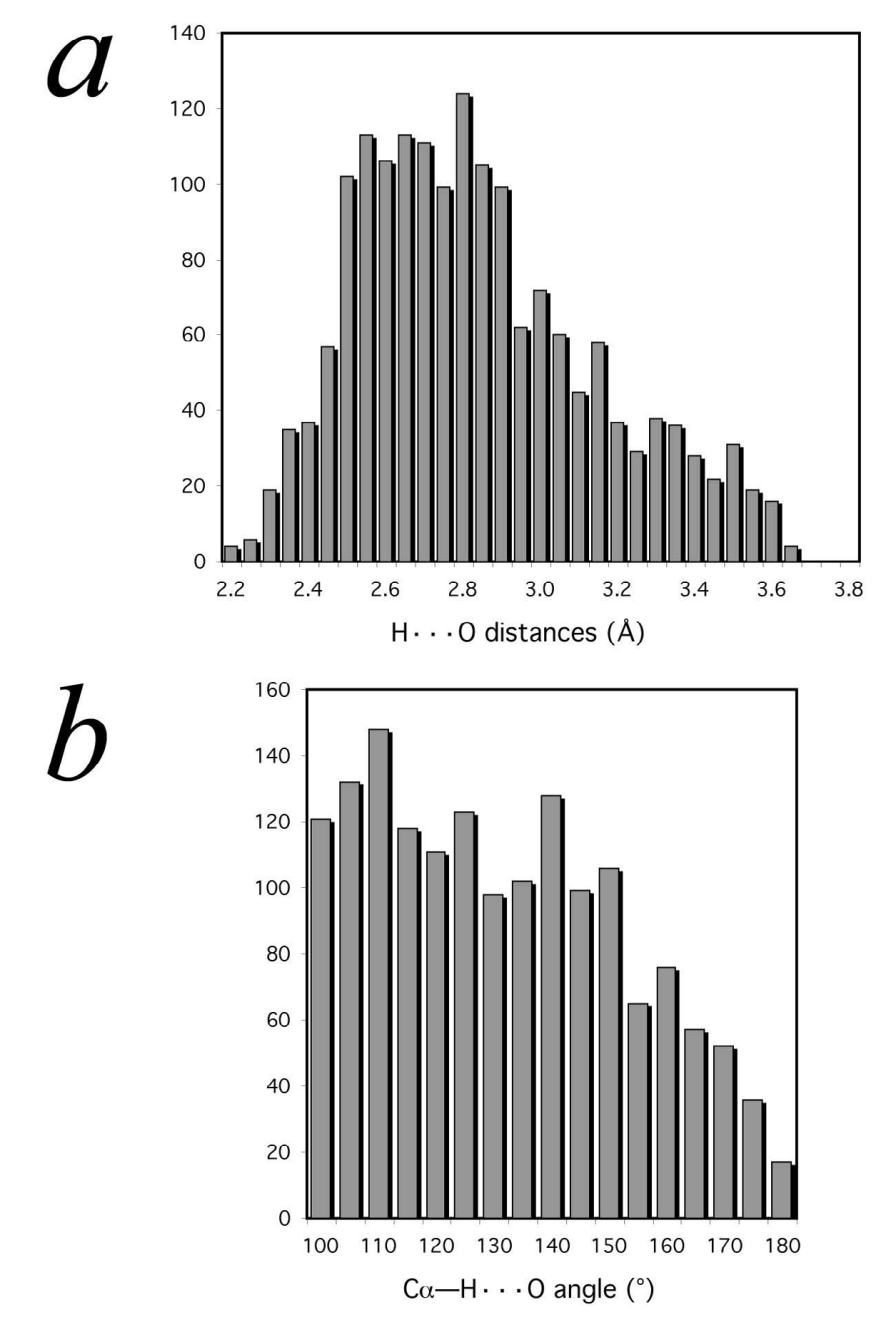
Distribution of H···O distances **(*a*) **and Cα-H···O angles **(*b*) **for the 1688 instances of single Gly-Cα-H···O hydrogen bonding in crystal structures of the Cambridge Structural Database (July 2003 release).

A similar level of confidence can be achieved for the Gly-Cα-Hα···O=C hydrogen bond by analysing the metrics of equivalent interactions in high-resolution crystal structures of small organic and organometallic molecules. Figure 3 shows the two fragment probes used in a statistical search for Gly-Cα-Hα···O=C nonbonded interactions in the Cambridge Structural Database (see Methods). Figure 4 shows that single hydrogen bonding (only one angle Cα-Hα···O ≥ 90°) predominates over the bifurcated case, and that a broad maximum in the Cα···O distribution occurs at about 3.4 Å, which can be taken as the "hydrogen bonding distance" for this type of interaction. This value is consistent with the theoretical value of 3.34 Å for the Gly-Cα-Hα···OH_2 _hydrogen bond from *ab initio *quantum calculations [22]. Figure 5 shows the distributions of Hα···O distances and Cα-Hα···O angles for the single hydrogen bond. The Hα···O distribution shows a broad maximum around 2.7 Å, whereas the angular distribution is very broad with maxima around 110° and 140°. Average parameters for single and double Gly-Cα-Hα···O=C hydrogen bonding (Table 1) are perfectly compatible with those calculated for the *c*RGD-αVβ3 and EFB-αIIbβ3 structures respectively, even though the accuracy of the values shown in Table 1 is clearly overestimated with respect to the resolution of these crystal structures. Thus, strictly from a geometrical point of view, the contacts between the Gly residues in the *c*RGD and EFB peptides and the main chain carbonyl group from Arg216 in the integrin surface bear all the characteristics of Cα-Hα···O=C hydrogen bonding. This observation is consistent with the exceptionally high frequency of intermolecular Gly-Cα-H···O=C hydrogen bonds recently reported in high resolution crystal structures of protein-ligand complexes [32].

**Figure 6:**
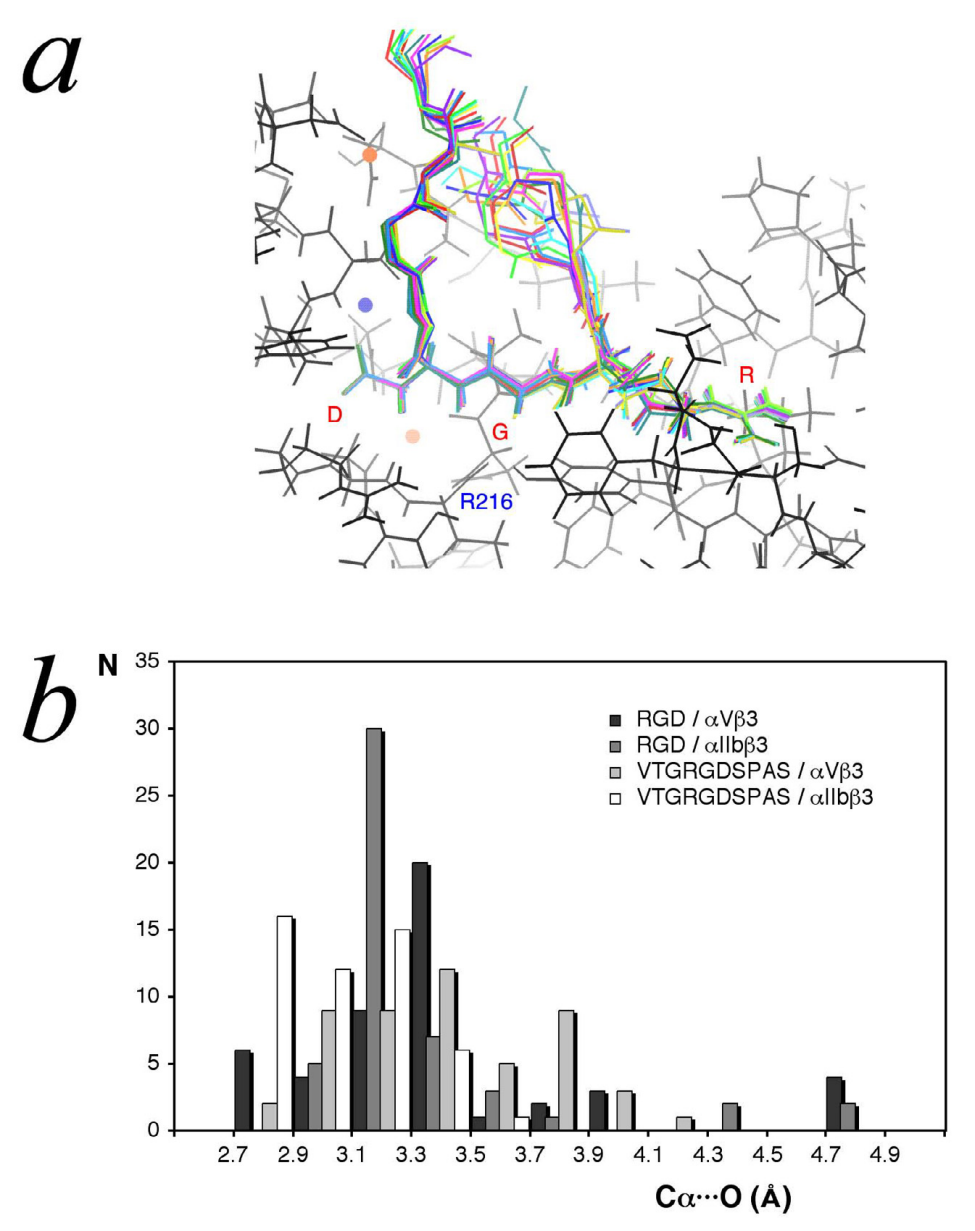
Results of molecular dynamics simulations of docking fibronectin RGD-containing peptides on to models of integrin surfaces. **(*a*) **Representation of the 15 lowest-energy models for the docking of the VTGRGDSPAS peptide on αIIbβ3 model surface. Integrin residues are shown in black whereas the 15 peptide models are shown in different colours. For peptide models, the only side chains shown are those from the RGD triad (indicated with red labels). In all models the central Gly residue in the RGD triad is located directly on top of the carbonyl group from Arg 216 in the β3 subunit (shown with blue label). **(*b*) **Distribution of Cα···O distances in the final models of the molecular dynamics simulations Distances computed between the carbonyl oxygen in Arg216 from the β3 subunit and the central Gly residue from the RGD triad.

A simple molecular docking analysis further supports the occurrence of Gly-Cα-H···O=C hydrogen bonds between RGD-containing ligands and integrin binding sites. In a first set of calculations, an RGD tripeptide was docked into the binding sites of both αVβ3 and αIIbβ3 integrins using constrained molecular dynamics (MD). Two constraints were imposed in the docking calculations: the carboxyl group from the Asp residue had to complete the metal coordination on the β3 subunit, and the Arg side chain had to form a salt bridge with appropriate Asp residues in the αV and αIIb subunits (see Methods), as observed in the crystal structures of αVβ3 and αIIbβ3 with different ligand-mimetics. Ten slightly different RGD models were obtained from the NMR structures of the adhesion domain of fibronectin [34], and were placed about 10 Å away from the integrin surface. Then these RGD models were subject to MD simulations until they docked into the integrin binding sites. In a second set of calculations, a longer peptide fragment with sequence VTGRGDSPAS from the adhesion domain of fibronectin was also docked into the binding sites of the two integrins (Figure 6). Again, ten different models for this peptide were obtained from fibronectin NMR structures [34]. Most of the simulations converged to models with Cα···O contact distances between the central Gly residue and the carbonyl of Arg216 in the 2.7–3.7 Å range (Figure 6b), with either one or two Gly-Hα atoms in hydrogen bonding orientation. These models were also the most favourable energetically (Table 2). From these calculations it seems to emerge that the RGD binding sites of the αVβ3 and αIIbβ3 integrins are primed to place the central Gly residue in the RGD triad directly above the carbonyl group of Arg216 of the β3 subunit (as observed in the *c*RGD-αVβ3 and EFB-αIIbβ3 crystal structures), forming one or two Cα-H···O hydrogen bonds that complement the main metal-coordination and salt-bridge interactions from the Asp and Arg side chains.

How important are these weak C-H···O hydrogen bonds in stabilising the *c*RGD-αVβ3 and EFB-αIIbβ3 complexes? A quantitative analysis of C-H···O hydrogen bonding at protein-protein interfaces has shown that they have an important contribution to the association and stability of protein complexes, accounting for about one third of the total hydrogen bonding interaction energy [27]. In fact, some of the hydrophobic or van der Waals interactions usually invoked to explain stabilising close contacts between molecules can be described better as weak C-H···O hydrogen bonds. These occupy a middle ground between the highly directional, conventional hydrogen bonds, and the directionless van der Waals interactions [32].

**Figure 7:**
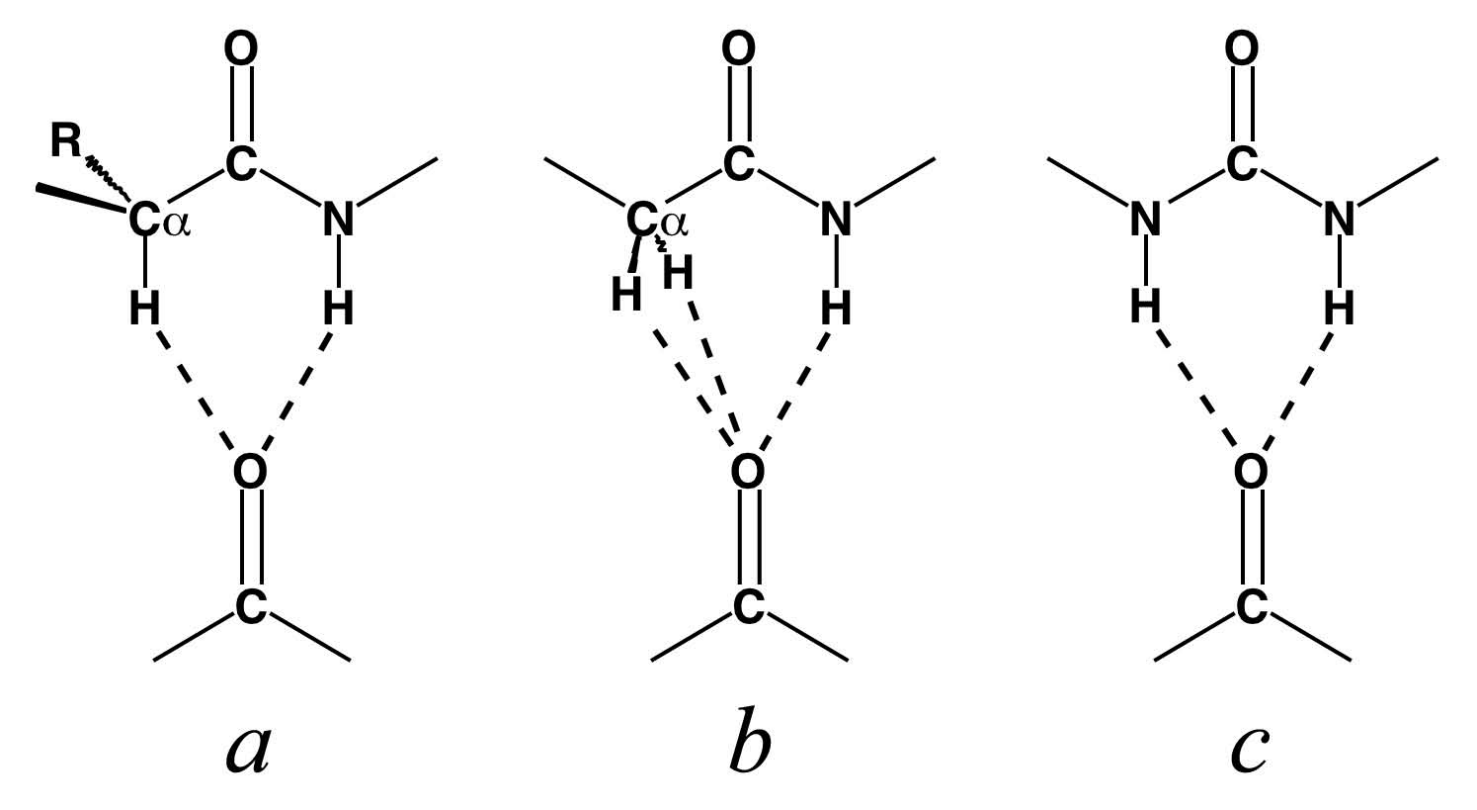
Bifurcated hydrogen bonding topologies. (***a***) The ubiquitous bifurcated hydrogen bonding topology seen in β-sheets and also in the collagen triple helix. The peptide chain is depicted with the *N*-terminus to the left. (***b***) Variation of the same bifurcated topology when the residue *N*-terminal to the donor N-H group is Gly, as observed in the *c*RGD-αVβ3 and EFB-αIIbβ3 crystal structures when Gly Hα atoms are built with standard geometry. Either one or two of the Gly Hα atoms can be in hydrogen bonding position. (***c***) Variation of the same bifurcated topology when the CH_2 _group *N*-terminal to the donor N-H group is replaced by another N-H group. This situation occurs for example when Gly is substituted by aza-glycine [37].

The recurrent appearance of some weak hydrogen bonding topologies in many structures of proteins and at protein-protein interfaces also reinforces the notion that they have a significant contribution to macromolecular stability. The most common occurrence of C-H···O hydrogen bonds in protein structures is a widespread Cα-H···O=C hydrogen bond *N*-terminal to the conventional N-H···O=C hydrogen bond in β-sheets [16,17] and in the collagen triple helix [15]. In this structural motif (Figure 7a), the Cα-H donor group is in the residue immediately *N*-terminal to the one carrying the N-H donor group, and both share the same C = O group as acceptor, an arrangement sometimes referred as "bifurcated" hydrogen bond [17,18,27]. This bifurcated hydrogen bonding motif is also the most common occurrence of C-H···O hydrogen bond at protein-protein interfaces [27]. The situation in Figure 7b occurs when the residue *N*-terminal to the one carrying the N-H donor group is Gly, with one or two Hα from Gly being in hydrogen bonding position. The bifurcated hydrogen bond scenario also occurs in the *c*RGD-αVβ3 and EFB-αIIbβ3 structures, where the N-H group from the Asp residue in the RGD peptide donates a hydrogen bond to the main chain carbonyl group from Arg216 of β3. This hydrogen bond has very bad geometry in the *c*RGD-αVβ3 structure (distance H···O 2.69 Å, angle N-H···O 133°), but looks better in the EFB-αIIbβ3 structure (distance H···O 2.39 Å, angle N-H···O 144°). These deviations from ideal hydrogen bonding geometry might be consequence of the resolution of the crystal structures, but all the MD docking simulations described above result in N-H···O=C hydrogen bonds that are slightly longer (typical H···O distances 2.4–2.5 Å) and slightly less linear (typical N-H···O angles 140°-150°) than the average hydrogen bonds between peptide groups in protein secondary structures. Automatic computational docking calculations of known integrin ligands on structural models of αVβ3 and αVβ5 consistently predict this N-H···O=C hydrogen bond to occur whenever an N-H group is present in the proximity of the carboxylate moiety [35,36]. Had these simulations included all nonpolar hydrogens, the companion Cα-H···O=C hydrogen bonds from the Gly residues would also have been observed.

**Figure 8:**
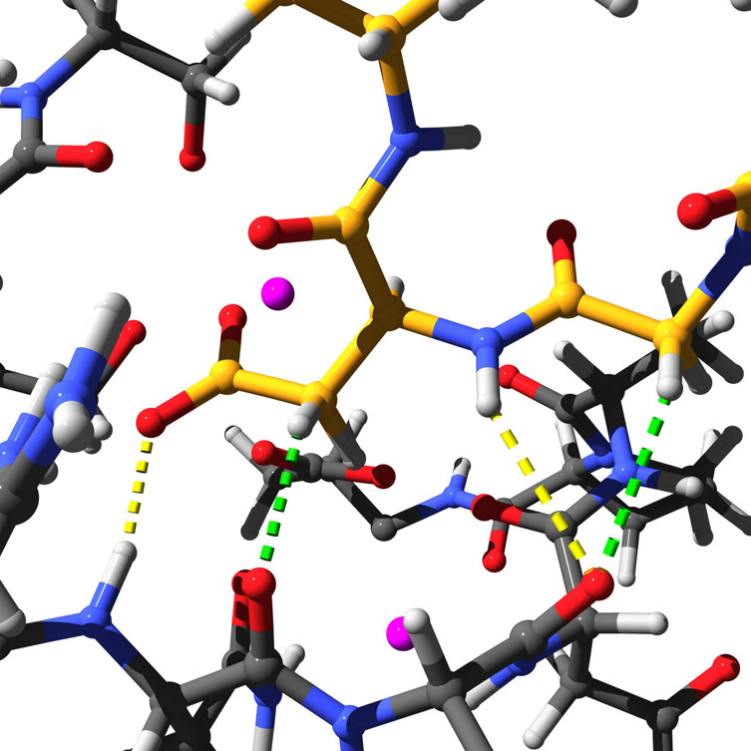
An array of four hydrogen bonds, two N-H···O=C (in yellow) and two C-H···O=C (in green), line up the bottom of the *c*RGD peptide against the integrin surface. The two weak Cα/β-H···O=C interactions thus contribute to the specificity of binding and presumably also have a cooperative effect on stability. Colour scheme for atom types as in Figure 2.

A quick inventory of hydrophobic interactions between the *c*RGD and EFB peptides and the αVβ3 and αIIbβ3 surfaces suggests an additional candidate for classification as C-H···O=C hydrogen bond, between the Cβ-Hβ group from the Asp residue of the *c*RGD and EFB peptides and the main chain C = O group from Asn215 in the β3 subunit. This weak hydrogen bond is adjacent to the stronger, conventional hydrogen bond between the main chain N-H group from Asn215 and Oδ2 from the Asp residue in the RGD motif. Thus, a total of four hydrogen bonds, weak and conventional, aligns the bottom of the *c*RGD and EFB peptides against the integrin surfaces (Figure 8), and complements the main interactions from the Asp carboxyl and Arg guanidinium groups to provide a higher binding specificity.

It is clear from the *c*RGD-αVβ3 and EFB-αIIbβ3 structures that any side chain other than Gly in the RGD triad would not allow it to fit snugly within the integrin binding site, with the resulting weakening of hydrogen bonding and van der Waals interactions. Furthermore the main chain conformation for the central Gly residue in the *c*RGD-αVβ3 structure falls in a region of the Ramachandran map that is not allowed to any L-amino acid residue. Thus, Gly residues at the centre of the RGD motif are essential for being small, for being able to adopt specific main chain conformations, and for being able to interact closely with the integrin surface via Cα-H···O=C hydrogen bonds. All three characteristics contribute to the integrin-binding specificity of Gly residues at the centre of RGD motifs.

Inasmuch as the *c*RGD-αVβ3 and EFB-αIIbβ3 structures remain valid models for the structural basis of integrin-RGD ligand-binding specificity, it is reasonable to assume that the weak Cα/β-H···O=C hydrogen bonds depicted in Figure 8 will also occur in RGD-based cell-adhesion interactions. A special feature of the integrin surface at the RGD-binding site is the presence of two main chain carbonyl groups exposed to the solvent in the β3 subunit: Asn215 and Arg216. In absence of ligands these groups will probably interact with water molecules through conventional hydrogen bonding interactions (as seen for example in the crystal structure of the cacodylate-bound form of αIIbβ3, PDB accession code 1TXV [12]). Upon ligand binding, the RGD residues will displace these waters and place one amide and two methylene groups in hydrogen bonding position to carbonyl groups, increasing the specificity of the RGD-integrin interaction through multipoint recognition (Figure 8). This strategy will obviously be exploited by many competitive inhibitors for the integrin RGD-binding site. For example it is possible to substitute the weaker Cα-H donors from the Gly residue by a conventional N-H group (Figure 7c). This strategy has been exploited already in the design of aza-peptide and azacarba-peptide RGD mimetics [37-39], several of them with nanomolar activity. Molecular modelling of the interaction of these peptides with αVβ3 and αVβ5 RGD binding sites predicts the hydrogen bonding topology shown in Figure 7c[36]. It is interesting to notice that even in the absence of a conventional hydrogen bonding donor, the carbonyl group Arg216 in the β3 subunit still may be acceptor for weak hydrogen bonds. In the crystal structure of αIIbβ3 in complex with tirofiban [12], a non-peptidomimetic inhibitor derived from L-tyrosine, the Cδ1 atom from the substituted Tyr ring is some 3.01 Å away from the carbonyl oxygen of the very same Arg216. If a hydrogen atom is built with standard geometry on Cδ1, the calculated Hδ1···O distance is 2.01 Å and the Cδ1-Hδ1···O angle is 172°, again hydrogen bonding-like metrics. How should this Cδ1···O=C contact be called? We think that a description in terms of weak C-H···O hydrogen bonding is in this case more accurate than referring to this interaction as simply hydrophobic.

## Conclusions

We have analysed in detail recently published structural data on the interaction between the extracellular regions of two integrins and peptides containing or mimicking the RGD sequence [8,12]. From this analysis we conclude that Cα-H···O=C hydrogen bonds from the central Gly residue also contribute to the specificity of binding. Weak hydrogen bonds are traditionally overlooked when describing protein structures, although they probably contribute to their stability. We think that our analysis provides one of the most interesting examples of C-H···O hydrogen bonds playing an important biological role, and may contribute to reverse the current trend of neglect of these interactions. In a recent paper, Sarkhel and Desiraju suggest that Nature may take advantage of the weaker C-H···O hydrogen bonds to optimise the efficiency of protein-ligand interactions, with a larger number of interactions coming into play even at the expense of the strength of the individual interactions [32]. By using more interactions, they suggest, specificity of recognition is increased, and because individual interactions are weaker, reversibility is possible. Our analysis of the interaction between the *c*RGD and EFB peptides and the αVβ3 and αIIbβ3 integrin surfaces would seem to corroborate this suggestion.

## Methods

### Integrin binding sites and hydrogen building

The following crystal structure coordinates were downloaded from the Protein Data Bank [40]: αVβ3 integrin in complex with a cyclic RGD peptide (*c*RGD), PDB accession code 1L5G [8]; αIIbβ3 integrin structure at 2.7 Å resolution, PDB accession code 1TXV [12]; αIIbβ3 in complex with eptifibatide (EFB), PDB accession code 1TY6 [12]; αIIbβ3 in complex with tirofiban, PDB accession code 1TY5. Models for integrin RGD-binding sites on αVβ3 and αIIbβ3 were obtained by selecting coordinates from integrin residues within 10 Å from the bound peptides. For the αIIbβ3 binding site, coordinates of the corresponding residues in the 1TXV structure were used, as this crystal structure has a better resolution. Coordinates for metal ions and structural waters present in the RGD-binding sites but not interfering with the binding of *c*RGD or EFB were also maintained. Hydrogen atoms were built with standard stereochemistry for the *c*RGD and EFB peptides and for the integrin RGD-binding sites as defined above, using the program *REDUCE *[41]. For the purpose of the analysis presented here all hydrogen atoms discussed in this paper could be positioned with satisfactory accuracy and predictable orientation.

### Molecular docking calculations

For the molecular docking calculations, conformational models for RGD and VTGRGDSPAS peptides were obtained from the NMR structures of the adhesion domain in fibronectin [34]. Ten conformational models were used for each peptide. Each model was first manually docked approximately into the coordinates of the binding sites of αVβ3 and αIIbβ3 integrins, using the *c*RGD-αVβ3 and EFB-αIIbβ3 structures for guidance. Then each docked model was pulled away to about 10 Å from the integrin surfaces, and was docked back into the integrin binding site via molecular dynamics (MD) simulations using the program *CNS *[42]. Five simulations were run for each model, to a total of 50 MD simulations for each peptide-integrin pairing. A set of distance restraints was applied to the docking MD simulations, as observed on the *c*RGD-αVβ3 and EFB-αIIbβ3 structures. The side chain of the Asp residue group was restrained to coordinate the bound metal ion in the RGD-binding sites and to receive a hydrogen bond from the amide group of Asn215, in the β3 subunit. The side chain of the Arg residue was restrained to form hydrogen bonds with residues Asp150 and Asp218 on the αV subunit or residue Asp224 on the αIIb subunit. Additional restraints were imposed in the MD simulations with the VTGRGDSPAS peptide: the ring of Pro172 was restrained to hydrophobic contact with the side chain of Lys125, in the β3 subunit, and the Cα atoms of the *N*- and *C*-terminal residues in the peptide model were restrained not to separate more than 5 Å from each other. The coordinates of the integrin binding sites were kept fixed in all the simulations, and only the peptides were allowed to refine by restrained MD and energy minimisation. All molecular models were analysed with the program CHAIN [43] in a Silicon Graphics workstation.

### Analysis of hydrogen bonding geometry in crystal structures of organic molecules

A survey in the Cambridge Structural Database [44] (July 2003 release), was carried out for Cα···O contacts between glycine-like fragments and carbonyl groups (Figure 3).

## List of abbreviations

ECM, extracellular matrix; RGD, Arg-Gly-Asp sequence; *c*RGD, cyclic pentapeptide with sequence Arg-Gly-Asp-D-Phe-N(Me)-Val; EFB, eptifibatide; PDB, Protein Data Bank; CSD, Cambridge Structural Database; MD, molecular dynamics.

## Authors' contributions

J.B. conceived the study and carried out the analysis of the structural data and molecular docking calculations. Both authors participated in the design, coordination and writing of the manuscript. Both authors read and approved the final manuscript.

## Figures and Tables

**Table 1:** Interatomic distances and angles for proposed and observed Gly-Cα-Hα···O hydrogen bonding interactions.

	PDB structures *			CSD Average values †	
	1L5G^*a*^	1TY6^*b*^	1CGD^*c*^	CH_2_···O	H-CH···O
*distances (Å)*					
Cα···O	3.39	3.48	3.15	3.45 (0.19)	3.44 (0.20)
Hα1···O	3.28	3.22	2.79	3.06 (0.25)	3.94 (0.38)
Hα2···O	2.68	2.99	2.63	3.05 (0.23)	2.81 (0.31)
*angles (°)*					
Cα–Hα1···O	86	94	100	106 (11)	50 (23)
Cα–Hα2···O	121	108	109	107 (11)	127 (23)
Hα1···O=C	136	139	110	118 (26)	119 (26)
Hα2···O=C	150	168	91	115 (27)	119 (24)

* Distances and angles measured on each structural model after standard-geometry Hα building on the central Gly residues. The accuracy of distances and angles in the integrin structural models is probably overestimated, due to the resolution of these structural determinations.

† Average values for instances of double (CH_2_···O) and single (H-CH···O) Gly-Cα-H···O hydrogen bonding in the Cambridge Structural Database (July 2003 release). Standard deviations in parentheses. See Methods and Figures 3, 4 and 5 for details of the search. In the single hydrogen bond case, column Hα 1 refers to the atom in hydrogen bonding position (Cα-Hα1···O ≥ 90°) and Hα 2 to the atom in non-bonding position (Cα-Hα2···O < 90°).

^*a *^RGD-αVβ3 crystal structure, PDB accession code 1L5G [8].

^*b *^EFB-αIIbβ3 crystal structure, PDB accession code 1TY6 [12].

^*c *^Average values from the crystal structure of the collagen-like peptide (Pro-Hyp-Gly)_4_-Pro-Hyp-Ala-(Pro-Hyp-Gly)_5_, PDB accession code 1CGD [15].

**Table 2:** Gly-Cα-H···O=C contact distances (Å) for the lowest-energy model in each set of molecular docking simulations. Underlined distances correspond to Hα atoms in hydrogen bonding orientation (angle Cα-H···O=C > 90°).

	Cα···O	Hα 1···O	Hα2···O
RGD-αVβ3	3.23	2.19	3.90
RGD-αIIbβ3	3.21	4.03	2.29
VTGRGDSPAS-αVβ3	3.31	2.96	2.73
VTGRGDSPAS-αIIbβ3	3.00	2.51	2.66
